# Accumulation of Bile in the Gallbladder: Evaluation by means of Serial Dynamic Contrast-Enhanced Magnetic Resonance Cholangiography with Gadolinium Ethoxybenzyl Diethylenetriaminepentaacetic Acid

**DOI:** 10.1155/2014/479067

**Published:** 2014-12-17

**Authors:** Tsutomu Tamada, Katsuyoshi Ito, Kazuya Yasokawa, Atsushi Higaki, Akihiko Kanki, Yasufumi Noda, Akira Yamamoto

**Affiliations:** Department of Radiology, Kawasaki Medical School, 577 Matsushima, Kurashiki City, Okayama 701-0192, Japan

## Abstract

The aim of this study was to evaluate the process of biliary excretion of gadolinium ethoxybenzyl diethylenetriaminepentaacetic acid (Gd-EOB-DTPA) into the biliary tract and to assess the accumulation patterns in the gallbladder using MR cholangiography obtained with Gd-EOB-DTPA which is a liver-specific hepatobiliary contrast agent. Seventy-five patients underwent Gd-EOB-DTPA enhanced MR imaging. Serial multiphasic hepatobiliary phase imaging was qualitatively reviewed to evaluate the process of the biliary excretion of contrast agent into the bile duct and the gallbladder. The accumulation pattern of contrast agent into gallbladder was classified into two groups (group 1 = orthodromic type and group 2 = delayed type). Furthermore, the results in differences of the presence of T1 hyperintense bile or sludge of gallbladder, gall stones, wall thickening of gallbladder, chronic liver disease, and liver cirrhosis between two groups were compared. Forty-eight of 75 patients (64%) were included in group 1, and remaining 27 (36%) were in group 2. The frequency of the presence of T1 hyperintense bile or sludge of gallbladder was significantly higher in patients with group 2 than that in patients with group 1 (*P* = 0.041). MR cholangiography obtained with Gd-EOB-DTPA showed that there may be an association between the biliary accumulation pattern in the gallbladder and the pathological condition.

## 1. Introduction

Assessment of the biliary excretion and bile accumulation in gallbladder including cystic duct patency might play an important role in determining appropriate treatment strategies for patients with biliary symptoms and in investigating the pathophysiology of gallbladder disorders such as cholecystolithiasis and acute or chronic cholecystitis.

Conventional T2-weighted MR imaging has been widely used as a noninvasive diagnostic modality to evaluate pancreaticobiliary disorders such as acute cholangitis and choledocholithiasis [1, 2]. However, it contains no dynamic functional information such as biliary excretion. On the other hand, MR cholangiography (MRC) with gadolinium ethoxybenzyl diethylenetriaminepentaacetic acid (Gd-EOB-DTPA) which is excreted into the bile ducts by a cellular process at the level of the hepatocyte can be used to obtain noninvasively high resolution postcontrast T1-weighted MR imaging, or so called functional MRC [3–11]. Until now, the functional MRC using Gd-EOB-DTPA has been used to evaluate cystic duct patency [3, 12, 13], biliary anatomy [3, 14, 15], relationship between the degree of biliary excretion and liver function [6, 16, 17], bile duct obstruction [18], bile duct injury including leakage and fistula [19–22], and biliary reflux [2]. However, the number of acquisitions of the multiphasic hepatobiliary phase (HP) imaging showing biliary excretion of Gd-EOB-DTPA in these studies excluding references 12 (unknown) and 15 (six phases) was 1 to 3. Accordingly, we hypothesized that dynamic contrast-enhanced- (DCE-) MRC using the multiphasic HP imaging acquired repeatedly in a short interval time can provide the further accurate information for the process of biliary excretion. To date, to our knowledge, few studies have focused on the accumulation of bile into the gallbladder using DCE-MRC with Gd-EOB-DTPA [3, 12, 13].

The aim of this study was to evaluate the process of biliary excretion of Gd-EOB-DTPA into the biliary tract and to assess the accumulation patterns in the gallbladder using DCE-MRC obtained with Gd-EOB-DTPA.

## 2. Materials and Methods

### 2.1. Subjects

Our institutional review board approved the review of patient medical records such as clinical and radiologic data for this retrospective study, and informed consent for the patients was waived.

Between March 3, 2011, and August 9, 2011, 117 consecutive patients underwent Gd-EOB-DTPA enhanced MRI of the liver for suspected liver diseases in our institution. 42 patients were excluded based on the following criteria: duplicate MRI examinations of the same patient (*n* = 7), patients with cholecystectomy (*n* = 19), patients without biliary excretion of Gd-EOB-DTPA to the extrahepatic bile ducts until 20 min after contrast agent administration (*n* = 2), patients with invisible gallbladder (*n* = 3), and a patient with direct invasion to common bile duct of gastric cancer (*n* = 1), incomplete examination such as lack of a part of the HP imaging (*n* = 5), and poor image quality (*n* = 5). A total of 75 patients (39 men, 36 women) with or without chronic liver disease (mean age: 65 years, range: 14–87 years) were thus included in this study. Sixteen patients without chronic liver disease had hepatic hemangioma (*n* = 1), hepatic metastasis (*n* = 6), portal vein thrombosis (*n* = 1), and no focal liver lesion (*n* = 8). In remaining 59 patients with chronic liver disease, the causes of chronic liver disease included type C viral hepatitis (*n* = 29), type B viral hepatitis (*n* = 11), alcohol abuse (*n* = 10), nonalcoholic steatohepatitis (*n* = 4), primary biliary cirrhosis (*n* = 1), autoimmune hepatitis (*n* = 1), and cryptogenic cirrhosis (*n* = 3). The chronic liver disease was diagnosed by established diagnostic criteria using medical history, blood tests, and/or liver biopsy. Thirty-three patients had cirrhosis and the remaining 26 did not. Among the patients with cirrhosis, 22 patients were graded as Child-Pugh class A and 11 patients as class B. Nineteen patients (25%) had gall bladder stone. No cases with other organic diseases of gall bladder such as acute cholecystitis and gall bladder cancer were included in this study.

Mean serum bilirubin and creatinine levels of all patients were 0.9 mg/dL (range: 0.3–2.5 mg/dL) and 0.72 mg/mL (range: 0.34–1.42 mg/dL), respectively.

### 2.2. MR Imaging Technique

Each examination was conducted with the subject in the supine position using a 1.5-T scanner (Signa Excite High speed; General Electric, Milwaukee, WI, USA, or EXCELART Vantage Powered by Atlas; Toshiba Medical Systems, Tochigi, Japan) equipped with a multichannel phased-array coil (4 channels phased-array torso coil; General Electric or Atlas SPEEDER Body combined with Atlas SPEEDER Spine (16 channels with 32 elements); Toshiba) for signal reception. Imaging was performed under fasting conditions in all patients.

Our routine MRI protocol included breath-hold in-phase and opposed-phase fast spoiled gradient-echo (GRE) T1-weighted imaging, breath-hold fast spin-echo (FSE) T2-weighted imaging with fat suppression, single shot FSE heavily T2-weighted imaging with fat suppression, breath-hold fluid-attenuated inversion-recovery imaging, diffusion-weighted imaging, and contrast-enhanced MR imaging (CE-MRI).

CE-MRI including vascular phase imaging (arterial, portal, and late phases) and HP imaging was performed before and after the administration of Gd-EOB-DTPA (Primovist; Bayer Schering Pharma, Osaka, Japan) by using a three-dimensional (3D) T1-weighted GRE sequence (TR/TE, 4.7-4.8/1.9–2.2 msec; flip angle, 12°–15° in transverse plane, 30° in coronal plane; parallel imaging factor, 2; FOV, 35 × 35 cm; slice thickness, 2.2–3.0 mm; matrix, 288–320 × 192–198) with fat-suppression technique (liver acquisition with volume acceleration [LAVA]; General Electric or Quick 3D; Toshiba) covering the whole liver. The acquisition time was 19-20 seconds. After the acquisition of unenhanced MR images, Gd-EOB-DTPA at a dose of 0.025 mmol/kg body weight was injected intravenously as a rapid bolus at a rate of 1 mL/sec with the use of a power injector (SONIC SHOT 50; NEMOTO KYORINDO, Tokyo, Japan) while the patient was in the bore of the magnet. This was followed by a 30-mL flush with normal saline (1 mL/sec). Among the CE-MRI, preenhancement imaging and multiphasic HP imaging were used for data analysis. HP images were obtained at 8, 10, 12, 14, 16, and 20 min in the coronal plane and 11, 15, and 21 min in the transverse plane after contrast media administration.

### 2.3. Image Analysis

Image evaluation was performed by two radiologists with 11 years and 7 years of experience in abdominal MR imaging in consensus using a PACS viewer. If the judgement of two reviewers was separated, another radiologist with 23 years of experience in abdominal MR imaging performed the final decision.

Serial HP imaging obtained in the coronal plane was qualitatively reviewed in each subject to evaluate the biliary excretion and the accumulation of bile in the gallbladder using a six-time-point scale after contrast media administration: timing point 1 = 8 min (HP1), timing point 2 = 10 min (HP2), timing point 3 = 12 min (HP3), timing point 4 = 14 min (HP4), timing point 5 = 16 min (HP5), and timing point 6 = 20 min (HP6). The excreted bile was defined as high signal intensity region in the hepatic duct, common bile duct, gallbladder and duodenum, and the accumulation pattern in the gallbladder were classified into two groups: group 1 (orthodromic type = visualization of Gd-EOB-DTPA into the gallbladder is earlier than that in the end of the common bile duct) and group 2 (delayed type = visualization of Gd-EOB-DTPA in the end of the common bile duct is earlier than that in the gallbladder). When visualization of Gd-EOB-DTPA in the gallbladder was simultaneous with that in the end of common bile duct, it was classified as group 1. When a high signal of Gd-EOB-DTPA was not visualized in both gallbladder and end of common bile duct by the timing point 6, such cases were not classified into this grouping because the accumulation pattern of these cases cannot be determined. Preenhancement images were used to assure that high signal intensity region in the gallbladder such as hyperintense sludge was not mistaken for contrast media. The presence or absence of contrast media in hepatic duct, gallbladder, common bile duct, and duodenum was evaluated using not only coronal HP imaging but also transverse HP supplementarily, and these HP images in different planes were cross-linked to each other on a PACS monitor.

Furthermore, for each subject, the presence or absence of (1) T1 hyperintense bile or sludge of gallbladder on preenhancement imaging and/or in-phase T1-weighted imaging, (2) gall stones on preenhancement imaging and/or T2-weighted imaging, (3) wall thickening of gallbladder of 4 mm or more on HP imaging, (4) chronic liver disease, and (5) liver cirrhosis was recorded and correlated with accumulation pattern of GD-EOB-DTPA in gallbladder. In addition, the diagnoses of sludge of gallbladder and gall stones were confirmed by preenhancement CT images and/or ultrasonography.

### 2.4. Statistical Analysis

Statistical analyses were performed using SPSS (version 19.0; SPPS, Chicago, Ill) software. The results in differences of the presence of (1) T1 hyperintense bile or sludge of gallbladder, (2) gall stones, (3) wall thickening of gallbladder of 4 mm or more, (4) chronic liver disease, (5) liver cirrhosis, and the distribution of (6) Child-Pugh classification between two groups were compared using Pearson's chi-square or the Fisher exact tests. Mann-Whitney *U* test was used to determine significant differences in the (7) serum bilirubin level two groups. *P* < 0.05 was considered statistically significant.

## 3. Results

### 3.1. Visualization of Biliary Excretion

The biliary excretion of contrast media in hepatic duct to common bile duct was successfully evaluated for all subjects in MRC obtained with Gd-EOB-DTPA.

### 3.2. Accumulation Pattern of Gd-EOB-DTPA in Gallbladder

**Figure 1:**
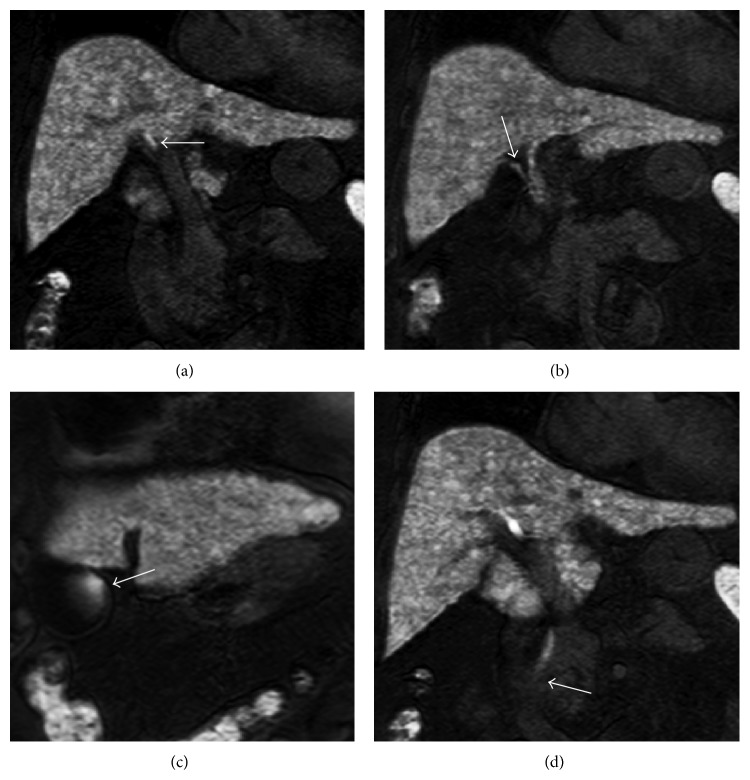
Orthodromic type of bile accumulation in gallbladder in a 72-year-old female with cryptogenic liver cirrhosis (Child-Pugh class-Pugh class A). MR cholangiography on coronal hepatobiliary phase (HP) imaging obtained after administration of Gd-EOB-DTPA demonstrates excretion of contrast media in common hepatic duct on 8 min (a) (arrow), cystic duct on 10 min HP image (b) (arrow), gallbladder on 12 min HP image (c) (arrow), and end of CBD on 16 min HP image (d) (arrow). This patient did not have stone, wall thickening, and T1 hyperintense bile in gallbladder (not shown).

**Figure 2:**
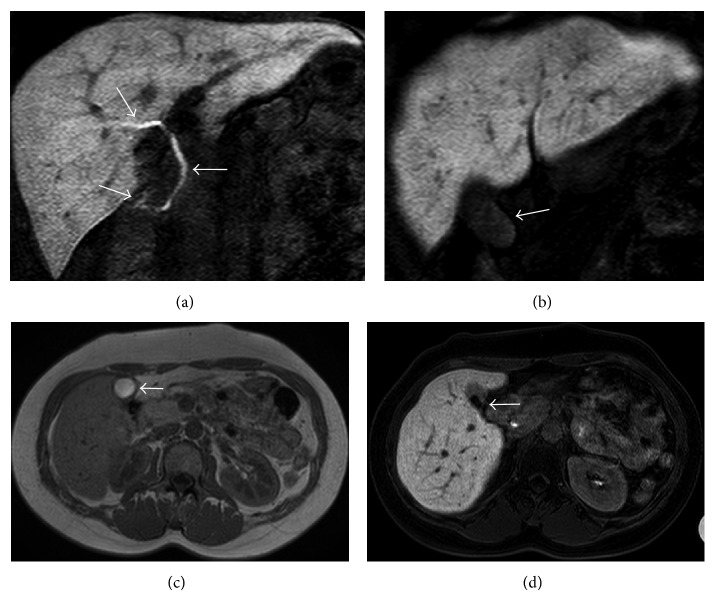
Delayed type of bile accumulation in gallbladder in a 37-year-old female with chronic hepatitis associated with type C viral hepatitis. On coronal hepatobiliary phase (HP) obtained 12 min after administration of Gd-EOB-DTPA, MR cholangiography demonstrates excretion of contrast media in intrahepatic duct to duodenum (a) (arrows). However, there is no accumulation of contrast media in gallbladder even in coronal 20 min HP image (b) (arrow). This patient had both T1 hyperintense bile on transverse in-phase fast spoiled gradient-echo T1 weighted image (c) (arrow) and gallbladder stone on transverse 20 min HP image (d) (arrow).

Of the 75 patients, two patients (3%) were not able to determine the accumulation pattern because the excretion of the contrast media in both gallbladder and end of common bile duct was not visualized by the timing point 6. Of the remaining 73 patients, 46 (63%) were included in group 1 (orthodromic type) (Figure 1), and remaining 27 (37%) were in group 2 (delayed type) (Figure 2). In 27 patients of group 2 (delayed type), as for 11 (41%), the excretion of contrast media to gallbladder was confirmed within 20 minutes after contrast media administration (delayed type-1), but 16 (59%) were not confirmed within 20 minutes after contrast media administration (delayed type-2).

### 3.3. Relationships between the Types of Accumulation of Bile in Gallbladder and Other Imaging and Clinical Findings

The frequency of the T1 hyperintense bile or sludge in gallbladder was significantly higher in patients of group 2 (delayed type) (19/27 (70%)) than that in patients of group 1 (orthodromic type) (21/46 (46%)) (*P* = 0.041). Furthermore, the T1 hyperintense bile or sludge in gallbladder was recognized in all patients (100%) of delayed type-1 and in 8 patients (50%) of delayed type-2. In addition, the frequency of gallstone in patients of group 2 (9/27 (33%)) was also higher than that in patients of group 1 (9/46 (20%)), but there was no significant difference (*P* = 0.188). The remaining five items showed no significant difference between two groups (Table 1).

## 4. Discussion

The process of biliary excretion into the bile duct could be assessed visually using functional MRC with Gd-EOB-DTPA obtained with multiphasic HP imaging. In experimental data, the 50% of Gd-EOB-DTPA is taken up in hepatocyte by active membrane transport system such as organic anion-transporting polypeptide (OATP) 8 and excreted into the bile duct via the export transporter such as multidrug-resistant protein (MRP) 2; the other 50% is distributed to the extracellular space and excreted into the kidneys [2, 17, 23]. The high excretion rate of this contrast agent to bile duct and the continuous acquisition of HP imaging by 3D T1-weighted GRE sequence with high contrast resolutions would result in the satisfactory visualization of the biliary excretion in functional MRC using Gd-EOB-DTPA.

In the present results for the accumulation of bile in the gallbladder, approximately two-thirds of patients were of orthodromic type, and remainder was delayed type. This result suggested that there are at least two kinds of inflow courses for the accumulation of bile in the gallbladder. Furthermore, orthodromic type was more frequent than delayed type as expected from the viewpoint of hydrodynamics.

Accordingly, if relationships between the accumulation pattern of bile in the gallbladder obtained with this study and biliary symptoms and pathophysiology of gallbladder disorders are clarified by further investigations, our observations suggest that DCE-MRC obtained with Gd-EOB-DTPA may aid in detecting the biliary disorders of early stage and in determining appropriate patient management including the treatment intervention and the critical prevention.

Presumed factors showing delayed type for bile accumulation of gallbladder include the association of (1) cystic duct patency, (2) increase of internal pressure and viscosity in gallbladder, and (3) decrease of excreted bile.

Of patients with delayed type, 59% (delayed type-2) might have cystic duct obstruction due to unknown cause since excretion of contrast media to gallbladder was not confirmed by the timing point 6 (the timing of 20 min HP imaging) unlike delayed type-1. However, these patients might have shown the accumulation of contrast media in the gallbladder after the timing point 6. Krishnan et al. have reported that gallbladder reflux of Gd-EOB-DTPA was demonstrated in 84% of patients by 20 min and in 92% of patients within 25 min after contrast administration [13].

Our results showed the relationship between increase of internal pressure and viscosity in gallbladder and delayed type in bile accumulation of gallbladder. That is, the frequency of T1 hyperintense bile or sludge in gallbladder was significantly higher in delayed type than in orthodromic type. Furthermore, 50% of the patients with delayed type-2 mentioned above had T1 hyperintense bile or sludge in gallbladder. Accordingly, it was suggested that several factors including the cystic duct patency and increase of viscosity in gallbladder may influence the delay of bile accumulation to gallbladder in the patients with delayed type-2 compositely. On the other hand, it was clarified that the increase of viscosity in the gallbladder rather than the cystic duct patency was strongly involved in delayed type-1 which recognized the T1 hyperintense bile or sludge of gallbladder in all patients. Furthermore, although it was expected that biliary dyskinesia also probably contributes to the hydrodynamics of bile, this study was not able to evaluate the biliary motor function [24].

In previous studies, it has been shown that the biliary excretion of Gd-EOB-DTPA was affected by the hepatocyte function [6, 16, 17]. Therefore, it is expected that the decrease of excretion of Gd-EOB-DTPA into the biliary tract in patients with impaired hepatic function such as liver cirrhosis may induce the delay of bile accumulation in gallbladder. However, our result was not able to show the association between patterns of bile accumulation in the gallbladder and presence or absence of hepatic dysfunction such as serum bilirubin level, cirrhosis, and chronic liver disease. In other words, it was indicated that functional MRC using continuous HP imaging with Gd-EOB-DTPA is able to assess the accumulation pattern of bile to gallbladder even in the patient with the liver dysfunction. However, because our study subjects did not have severe liver cirrhosis like Child C, the contribution of certain bile accumulation patterns in the gallbladder to liver dysfunction might not be accurately evaluated.

There were some limitations to our study. First, the number of subjects was relatively small. Therefore, a study with large patients is needed to confirm the results obtained at this study. Second, in our study, HP imaging was acquired up to 20 min after contrast media administration. Therefore, it is unknown whether patients who were considered to have the obstruction of cystic duct showed the accumulation of contrast media in the gallbladder on HP imaging greater than 20 min. However, of 75 patients in this study, the relationship of biliary excretion between gallbladder and common bile duct could be assessed in 73 patients (97%) using HP imaging up to 20 min. Third, since HP imaging was acquired at 2- to 4-minute intervals, the relatively long intervals might have a potential of missing the detection of the contrast media that rapidly flows through the common bile duct in the orthodromic direction. Fourth, we might assess the accumulation of bile in the gallbladder using a noninvasive MR technique such as non-contrast-enhanced cine-dynamic MRC with a spatially selective IR pulse [25]. Finally, we have not evaluated the relationship between the pattern of accumulation of bile in the gallbladder and the pathophysiology of gallbladder disorders. Further clinical studies in patients with various gallbladder disorders such as acute or chronic cholecystitis are needed to clarify the influence on the treatment strategies for patients with biliary symptoms in accordance with patterns of bile accumulation in the gallbladder using MRC with Gd-EOB-DTPA.

In conclusion, the accumulation of bile in the gallbladder could be assessed visually with MRC using Gd-EOB-DTPA. Furthermore, the delay in the bile accumulation in the gallbladder is associated with T1 hyperintense bile or sludge in gallbladder and gallstones, indicating the increase of viscosity and pressure in the gallbladder. Accordingly, there may be an association between the biliary accumulation pattern in the gallbladder and the pathological condition.

## Figures and Tables

**Table 1:** Relationships between the type of accumulation of bile in gallbladder and each item of imaging findings and clinical conditions.

Item	Orthodromic type	Delayed type	*P* value
T1 hyperintense bile or sludge^*^	46% (21/46)	70% (19/27)	0.041
Gallbladder stones^*^	20% (9/46)	33% (9/27)	0.188
Gallbladder wall thickening > 4 mm^*^	11% (5/46)	4% (1/27)	0.403
Chronic liver disease^*^	76% (35/46)	81% (22/27)	0.591
Liver cirrhosis^*^	41% (19/46)	44% (12/27)	0.793
Child-Pugh classification	Child A 12/Child B 7	Child A 9/Child B 3	0.697
Serum bilirubin level (mg/dL)^†^	0.94 ± 0.51	0.87 ± 0.47	0.571

^*^Data represent percentages, with values used to calculate these percentages provided in parentheses.

^†^Data are given as mean ± standard deviation.
